# CRISPR-based rapid and ultra-sensitive diagnostic test for *Mycobacterium tuberculosis*

**DOI:** 10.1080/22221751.2019.1664939

**Published:** 2019-09-15

**Authors:** Jing-Wen Ai, Xian Zhou, Teng Xu, Minling Yang, Yuanyuan Chen, Gui-Qing He, Ningp Pan, Yuwei Cai, Yongjun Li, Xiaorui Wang, Hang Su, Ting Wang, Weiqi Zeng, Wen-Hong Zhang

**Affiliations:** a Department of Infectious Diseases, Huashan Hospital, Fudan University, Shanghai, People’s Republic of China; b Vision Medicals Co., Ltd, Guangzhou, People’s Republic of China; c Tuberculosis Diagnosis and Treatment Center, Hangzhou Red Cross Hospital, Hangzhou, People’s Republic of China; d Department of Infectious Diseases, Wenzhou Central Hospital, Wenzhou, People’s Republic of China; e Department of Infectious Diseases, Jing’an District Central Hospital of Shanghai, Shanghai, People’s Republic of China

**Keywords:** CRISPR-MTB, tuberculosis, *Mycobacterium tuberculosis* complex (MTB), GeneXpert MTB/RIF, diagnosis

## Abstract

Rapid and simple-to-use diagnostic methods for tuberculosis are urgently needed. Recent development has unveiled the diagnostic power of the CRISPR system in the detection of viral infections. However, its potential use in detecting the *Mycobacterium tuberculosis* complex (MTB) remained unexplored. We developed a rapid CRISPR-based assay for TB detection and conducted a retrospective cohort study of 179 patients to evaluate the CRISPR-MTB test for identifying MTB in various forms of direct clinical samples. Its diagnostic performance was compared, in parallel with culture and the GeneXpert MTB/RIF assay (Xpert). The CRISPR-MTB test is highly sensitive with a near single-copy sensitivity, demands less sample input and offers shorter turnaround time than Xpert. When evaluated in the clinical cohort of both pulmonary and extra-pulmonary tuberculosis, the CRISPR-MTB test exhibited an overall improved sensitivity over both culture (79% vs 33%) and Xpert (79% vs 66%), without comprise in specificity (62/63, 98%). The CRISPR-MTB test exhibits an improved overall diagnostic performance over culture and Xpert across a variety of sample types, and offers great potential as a new diagnostic technique for both pulmonary and extra-pulmonary tuberculosis.

## Introduction

Tuberculosis (TB) is the leading cause of death among infectious diseases, with higher mortality than HIV and malaria worldwide [1]. To a great extent, this is due to the difficulty in its diagnosis. It was estimated that 40% of the cases failed to be identified and reported [1]. The GeneXpert MTB/RIF assay (Cepheid Inc, Sunnyvale, CA, USA) (hereinafter referred to as “Xpert”) was endorsed by the World Health Organization in 2010 [2]. However, a recent study showed that the Xpert test had not improved global detection rates and showed limited efficacy in diagnosing extra-pulmonary tuberculosis [3]. As rapid and accurate diagnosis of tuberculosis is a prerequisite for effective treatment, alternative methods which allow rapid screening and diagnostic are urgently needed.

Clustered regularly interspaced short palindromic repeat (CRISPR)-associated proteins (Cas) are adaptive immune systems of archaea and bacteria [4–6]. Cas9 was the first member of the Cas family which had been used for gene editing since 2013 [7,8]. In the last couple years, by taking advantage of their collateral, promiscuous cleavage activities, a unique group of Cas enzymes including Cas12, Cas13 and Cas14, were harnessed for *in vitro* diagnosis of viral pathogens such as ZIKA and Dengue [9–11]. All three Cas-enzymes shared similar reactive components, which include gRNA, probes, nucleotide activators, buffers and itself. They all have two distinct enzymatic domains. One of the domains binds to nucleotide activator and another one cleave small nucleotide probes when it gets activated. Cas12 recognizes double-strand DNA (dsDNA) as activator and cleave single-strand DNA (ssDNA) [9]. Cas13 recognizes ssRNA and cleave ssRNA[10]. Cas14 recognize ssDNA and cleave ssDNA [11]. Protospacer adjacent motif (PAM), which is a 2–6 base pair DNA sequence immediately following the DNA sequence targeted by the Cas12 nuclease, is required for processing of target [9]. Importantly, when combined with a DNA amplification step, these CRISPR systems could detect target nucleic acid molecules at the sensitivity of as low as 5 aM^10^. A few other studies further extended their applications on identifying a variety of other viruses, suggesting their great potential in the diagnosis of viral infections[12–14]. However, the application of the CRISPR systems in bacterial diagnosis, such as the *Mycobacterium tuberculosis* (MTB) complex remained largely undetermined.

Here, we conducted a retrospective, comparative study aiming to compare the diagnostic performance of MTB among different technology platforms including CRISPR-based MTB test (referred to CRISPR-MTB hereafter), Xpert, and culture. For a more comprehensive evaluation, we collected clinical samples in multiple forms, including sputum, bronchoalveolar lavage fluid (BALF), cerebrospinal fluid (CSF), pleural fluid, ascites, pus, etc., which were either subjected to culture or directly tested in the Xpert and our CRISPR-MTB assays.

## Materials and methods

### Study participants and samples collection

We retrospectively reviewed all 317 patients admitted into Huashan Hospital with suspicion of active MTB infection, including both pulmonary TB and extrapulmonary TB, by their attending physicians from 1 June 2017 to 30 June 2019. As a tertiary hospital, Huashan Hospital admitted patients from Shanghai and all over the country who were referred from local hospitals. Sample collection was reviewed and approved by the Huashan Ethics Committee Review Board. Informed consents were signed by patients or surrogates. Samples were prepared and gathered from suspected infected parts of enrolled patients and all clinical specimen were sent for culture, Xpert and other microbiological assays if necessary. All the samples were stored at −80°C before CRISPR-MTB. For patients suspected for pulmonary TB, at least two sputum specimens were sent for MTB culture. All samples were collected either prior to or within 7 days of anti-TB therapy. The Xpert assay was performed following the manufacture instructions. Exclusion criteria included (1) incomplete clinical data, (2) incomplete microbiological data, (3) insufficient specimen for CRISPR-MTB. A total of 179 samples were included for this study. Final clinical diagnosis of the enrolled cases was made by the attending physicians according to China Clinical Treatment Guide for Tuberculosis [15] and other clinical guidelines. Patients with MTB culture-positive or Xpert-positive results for MTB would be classified as microbiologically confirmed TB cases. For patients without microbiological evidence, attending physician may only clinically diagnose active MTB infection by combining the patient's clinical manifestations and imaging findings to exclude other diseases, together with the patient’s confirmed responsiveness to anti-TB treatment after one month of follow-up.

### Samples classification

Samples were categorized as MTB cases and non-MTB cases. The MTB group included pulmonary TB and extrapulmonary TB. The non-MTB group included infections other than TB, malignancies, noninfectious inflammatory diseases and miscellaneous causes.

### Cas12a protein and other reagents

The open reading frame (ORF) of Cas12a was synthesized by GenScript (Scotch Plains, NJ) following a sequence optimized for expression [9]. The Cas12a ORF was then cloned into expression vector and transfected into *E. coli* BL21. *E. coli* cells were first grown at 37°C followed by incubation with IPTG at 16°C for Cas12a expression. Proteins were purified from lysed bacteria using the Ni-NTA protocol. Purified proteins aliquots were stored at −80°C. Other reagents were purchased from Sangon Co., Ltd. (Shanghai, China), including DTT (A100281), EDTA (A100105), TritonX-100 (A110694), NP-40 (A100109), Chelex-100 (C7901) etc.

### Strains and human DNA

*M. tuberculosis* H37Ra strain was purchased from the American Type Culture Collection (ATCC25177). *M. bovis* (BCG) and several species of Nontuberculous mycobacteria (NTM), including, *M. kansasii*, *M. abscessus*, *M. avium*, and two species of bacteria, *E. coli* and *S.agalactiae* were purchased from China General Microbiological Culture Collection Center (CGMCC). Pure human DNA were purchased from Solarbio Co., Ltd. (Beijing, China), and eluted in nuclease-free water.

### Oligos and gRNA

So far, there have been many well studied, conserved target regions for MTB, which include rpo B, kat G, inh A, IS6110, IS1081, etc. [16,17]. These genetic regions have also been commonly used for the development of clinical *in vitro* diagnostic products for MTB. When developing CRISPR-MTB in this study, we decided on IS6110 based on that there are multiple copies of IS6110 per MTB genome, which may be advantageous for assay sensitivity.

Primer sets, including forward primer 5′-GGTC GGAA GCTC CTAT GACA ATGC ACTA GCC and reverse primer 5′-TTGA GCGT AGTA GGCA GCCT CGAG TTCG AC, was synthesized for the amplification of the IS6110 region for MTB detection. There are 17 complete copies of IS6110 in H37Ra [17]. gRNA 5′-UAAU UUCU ACUA AGUG UAGA UAUC AGCU CGGU CUUG UAUA G adjacent to PAM sequence(TTTG) and oligo probe 5′-6-FAM-TTTT TTTT TTTT-BHQ1 were used in this study.

### DNA rapid extraction

Sputum were mixed with 2 volumes of liquefaction buffer, which consists of 0.1% DTT, 0.78% NaCl, 0.02% KCl, 0.02% KH_2_PO_4_, and incubated at 37°C for 30 min. Five hundred microlitre treated sputum or other sample was transferred to a new sterile, nuclease-free 1.5 ml tube. After 5 min of centrifugation at 10,000 g, the pellet was resuspended in lysis buffer, which consists of 0.1% SDS and 1% NP40. Glass microbeads with 0.1–0.2 mm diameter were added at 25 mg per tube. Vortex Mixer (Crystal, Texas) were used to break the bacterial cell wall at 3000 rpm for 5 min. Tubes were then heated at 99°C for 10 min followed by 10 min of centrifugation at 14,000 g. Finally, 2.5 μl of supernatant will be used as a template for subsequent experiments.

### CRISPR-MTB assay

The CRISPR-MTB test combines an Recombinase Polymerase Amplification (RPA) step and a following Cas12a detection step in the same reaction as described previously [9]. Briefly, 25 µl reactions containing 2.5 µl sample, 0.4 µM forward and reverse primer, 1× reaction buffer, 14 mM magnesium acetate and RPA mix were incubated at 37°C for 30 min. After that, all the amplification product was then added to the CRISPR reaction mix consisting of 33.3 nM of gRNA, 66.7 nM of Cas12a and 166 nM of ssDNA reporter. This final reaction was incubated at 37°C and monitored for fluorescence signal for 20 min. Fluorescent signals were collected by an ABI7500 qPCR machine (Thermo Fisher, Massachusetts) for the duration of 20 min.

### Evaluation of LOD (limit of detection)

We evaluated LOD in both DNA and quantified CFU. The reference strain of MTB, H37Ra, was revived and amplified by culturing (MGIT, Becton-Dickinson, Heidelberg, Germany). Serial dilution assay was performed to determine the number of viable bacterial. Viable MTBs more than 10^8 ^CFU/mL were used in this study. Indicated number of MTBs were added into negative sputum samples and subjected to our optimized procedure for LOD determination. Total eight doses were tested, and 10 repeats were performed for each dose. The dose point with 100% detection rate was taken as LOD. DNA was purified from H37Ra strain by using column-based extraction kit, and quantified with Qubit assay. DNA copies number were determined following formula (6.02 × 10^23^) × (ng/μl × 10^−9^)/(DNA length × 660) = copies/μl. DNAs were diluted serially as indicated in results. 2.5μl DNA from each diluted solution were used as templates. 10 repeats were performed at every dose point.

### Data procession and statistical analysis

Equal *M. kansasii* strain DNA(50,000copies) was used as negative control (NC). The samples yielded 2.0 or more folds of fluorescent signal above NC were determined as positive. The *t*-test and Fisher’s exact test were employed to evaluate continuous and binomial variables, respectively. The results were presented with the range of 95% confidence intervals. We did analysis of variance test to compare differences across subgroups. Statistical analyses and figures were conducted using the SPSS statistical package 22.0 software and GraphPad Prism 5 software.

## Results

### Development of the CRISPR-MTB assay

**Figure 1.**
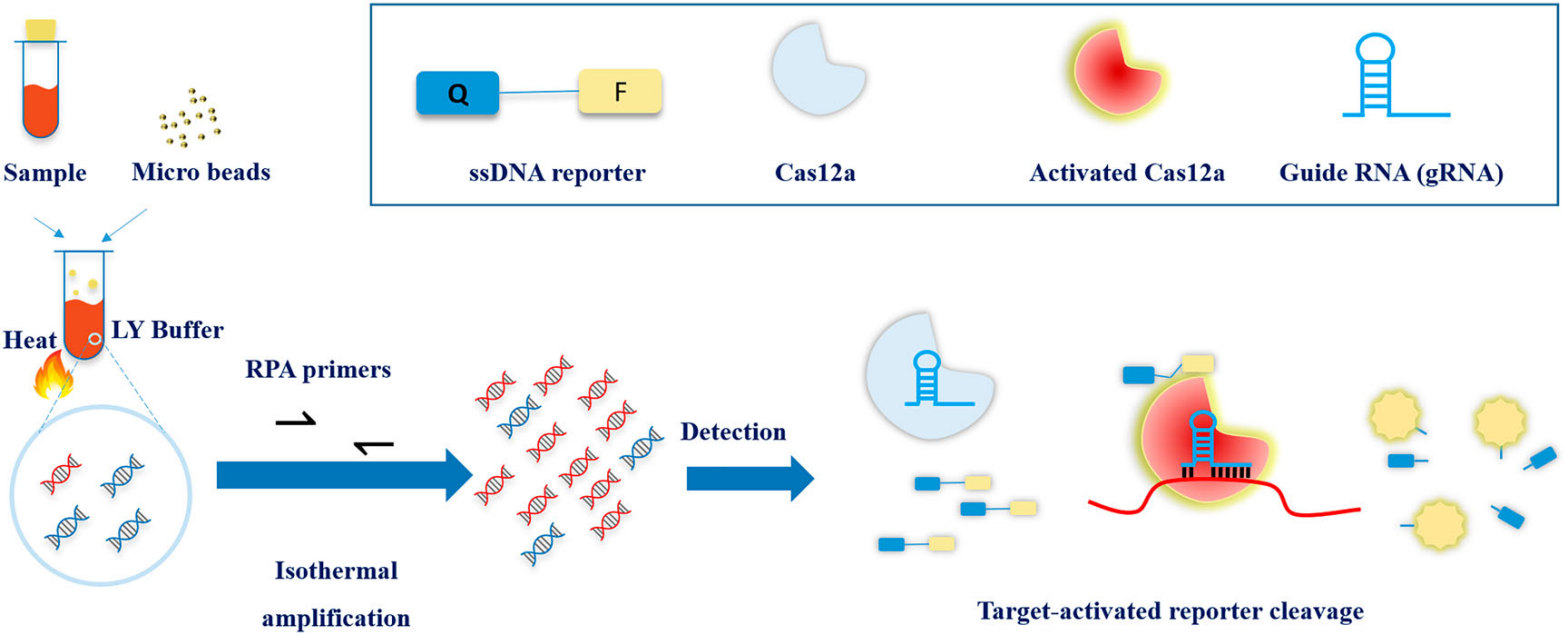
Scheme of CRISPR-MTB. Samples were added into LY buffer with microbeads, and were vortexed/heated. Supernatants were subjected to CRISPR-MTB system. Positive fluorescent signals were captured when probes were cleaved by activated Cas12a under target sequence recognized by gRNA.

We developed a rapid, highly sensitive and simple-to-use MTB assay by combining a RPA reaction with a CRISPR/Cas12a step for target detection (Figure 1). We chose IS6110, an MTB-specific insertion sequence of ∼1.5 kb in length as the target sequence in this assay for the reason that there are 6–10 copies per genome and therefore, would render higher molecular sensitivity for MTB detection. Multiple sets of RPA primers and CRISPR gRNA targeting different regions within IS6110 were screened (data not shown). The set that showed the best overall performance of sensitivity and specificity was then used throughout this study for assay optimization and clinical diagnostic evaluation.

**Figure 2.**
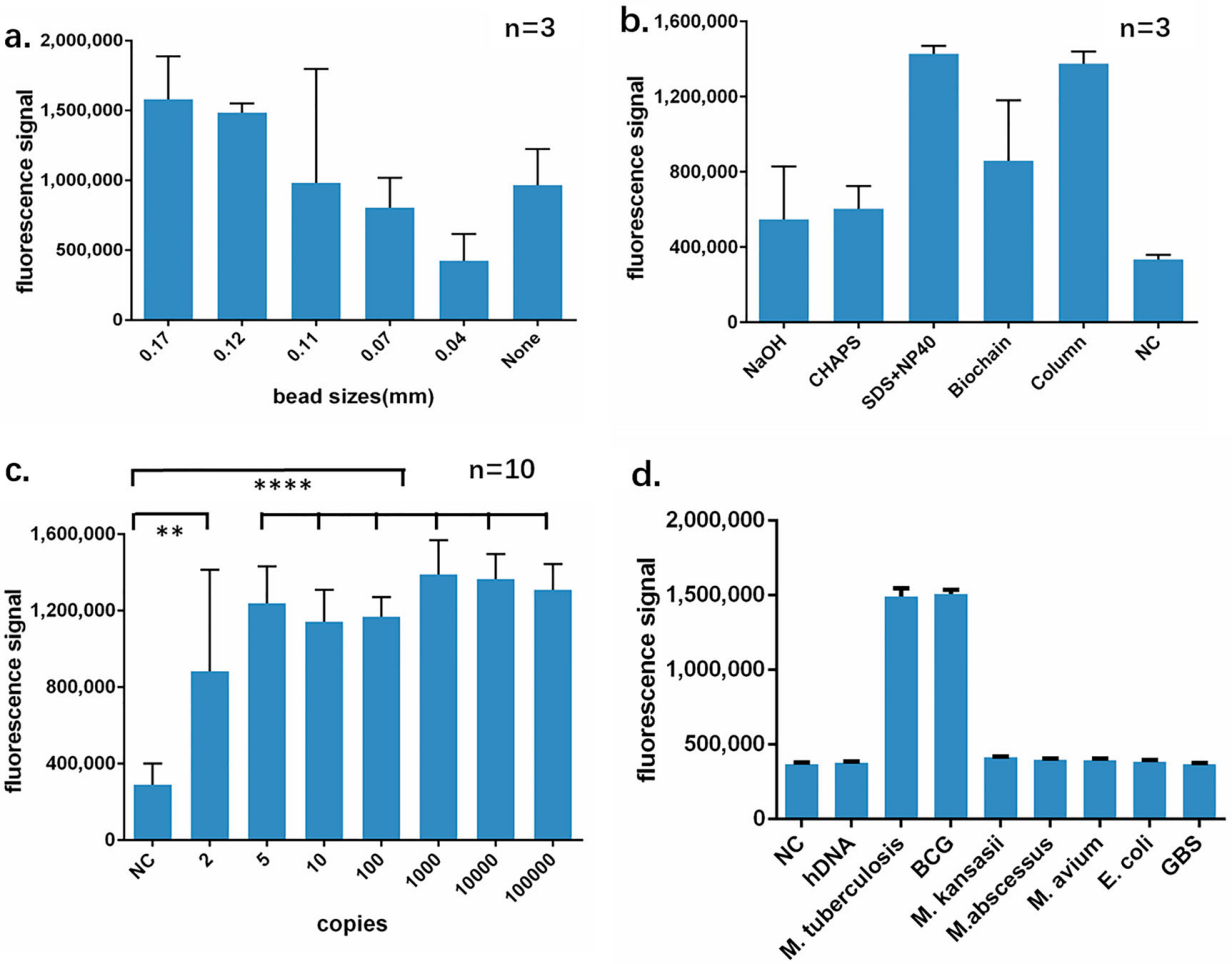
Performance identification of CRISPR-MTB. (a) Diameters of the beads between 0.17 and 0.11 mm are suitable for MTB broken. Each type of beads was added into lyse buffer followed same extraction and CRISPR process. No beads were served as negative control. (b) MTB DNA from optimized recipe of rapid extraction yields similar signal comparing to DNA purified by column. All three rapid extraction recipe shared same component including EDTA and Tris with indicated NaOH, CHAPS or SDS + NP40. Commercially available rapid extraction kit from Biochian was compared. DNA from column-based extraction obtained by Sangon bacteria genomic DNA purification kit was served as positive control. DNA from M. kansasii strain was rapidly extracted by SDS + NP40 and served as negative control. (c) CRISPR-MTB can detect MTB DNA close to single copy level. (d) CRISPR-MTB yields specific signal from MTB and BCG but no other DNAs. All bacteria were purified as standard manual of bacteria genomic DNA purification kit. 5 ng genomic DNA of bacteria or 50 ng hDNA was subjected to CRISPR-MTB. ** *P* < 0.005, **** *P* < 0.0001

To establish a rapid and simple-to-use extraction method for the CRISPR-MTB assay, we set out to optimize the extraction process based on a combination strategy of beads beating, chemical lysis and heating to ensure high efficiency of MTB DNA extraction. We first optimized the size of the glass beads in order to achieve optimal breaking of the cell walls. Beads with a serial of diameters from 0.04 to 0.17 mm were tested. We found that beads with diameters of 0.11–0.17 mm offered the greatest detection signal whereas those of 0.04 or 0.07 mm showed limited or little effect when compared to a beads-free protocol (Figure 2(a)), suggesting that selection of the bead size is crucial for MTB extraction. We further optimized the chemical lysis process and found that the lysis buffer consisting of 0.1% SDS and 1% NP40, delivered better extraction results than other lysis conditions as determined by the fluorescence output from the CRISPR assay (Figure 2(b)). The classical column-based DNA protocol assay has been recognized as an efficient way of DNA extraction. By comparing our optimized extraction protocol to the column-based method, we observed comparable assay sensitivity as indicated by similar fluorescence readings. These results suggest an uncompromised extraction efficiency of our extraction method, with a reduced extraction time and fewer centrifugation steps over column-based traditional method.

To determine the analytical sensitivity of the CRISPR-MTB assay, we serially diluted DNA from MTB strain H37Ra for the evaluation of LOD. Equal volume of TE buffer was used as negative control. We found that the CRISPR assay could consistently detect 5 copies/ul of MTB DNA in10 out of 10 tests, and 2 copies/ul in 6 out of 10 tests, suggesting that the LOD in DNA could reach 5 copies/ul level, showing an ultra-high sensitivity close to single copy level (Figure 2(c), Fig. S1). When further evaluating the LOD in CFU, we found CRISPR-MTB assay detecting the LOD of 50 CFU/ml (Fig. S2).

In contrast to the substantial signal produced in the presence of Mycobacterium tuberculosis complex (MTB and Bacillus Calmette–Guérin), none of the DNA originated from human, NTM(*M. kansasii, M. abscessus and M. avium)* or other bacteria (*E. coli* and *S. agalactiae*) triggered false positive reaction (Figure 2(d), Fig. S3). These data suggest the CRISPR-MTB as a highly sensitive and specific assay for MTB detection.

### Application of CRISPR-MTB in clinical tuberculosis

**Figure 3.**
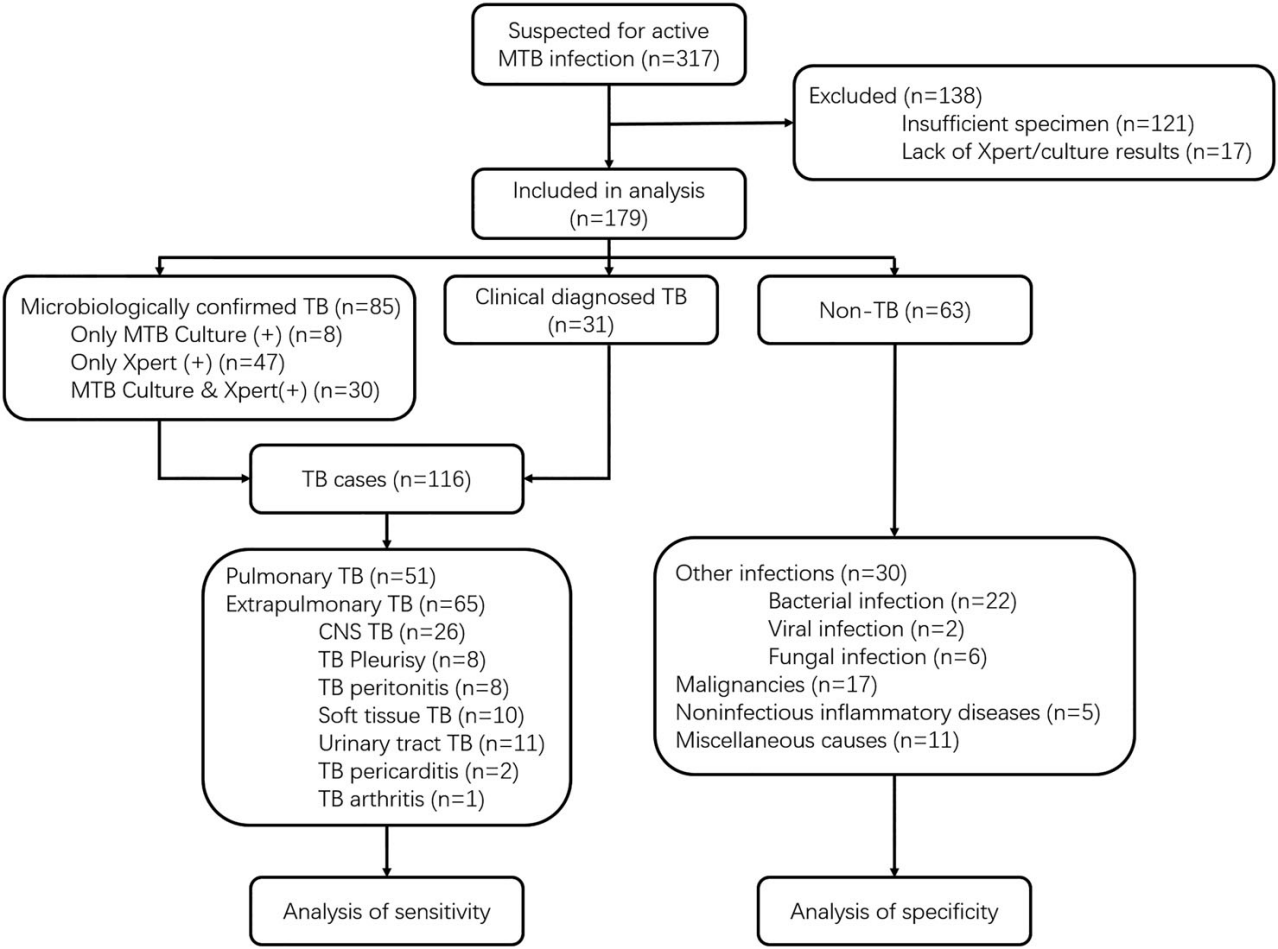
Study design.

After establishing the CRIPSR-MTB assay, we set out to evaluate its diagnostic potential in clinical tuberculosis. From 1 June 2017 to 30 June 2019, 317 patients presenting with suspected active MTB infection were reviewed and considered as the cohort for this study. Among these patients, 138 were excluded because of failure to achieve required microbiological test results and/or insufficient specimen for CRISPR-MTB testing. The remaining 179 patients were all tested for MTB by culture, Xpert and later CRISPR-MTB retrospectively on direct clinical samples (Figure 3). 116 of 179 participants had clinical final diagnosis of active MTB infection, including 51 pulmonary TB cases and 65 extrapulmonary TB cases. Patient characteristics of those with and without active TB infection did not differ aside from age, sexuality proportion and blood T.SPOT TB results (supplemental materials, Table S1).

**Figure 4.**
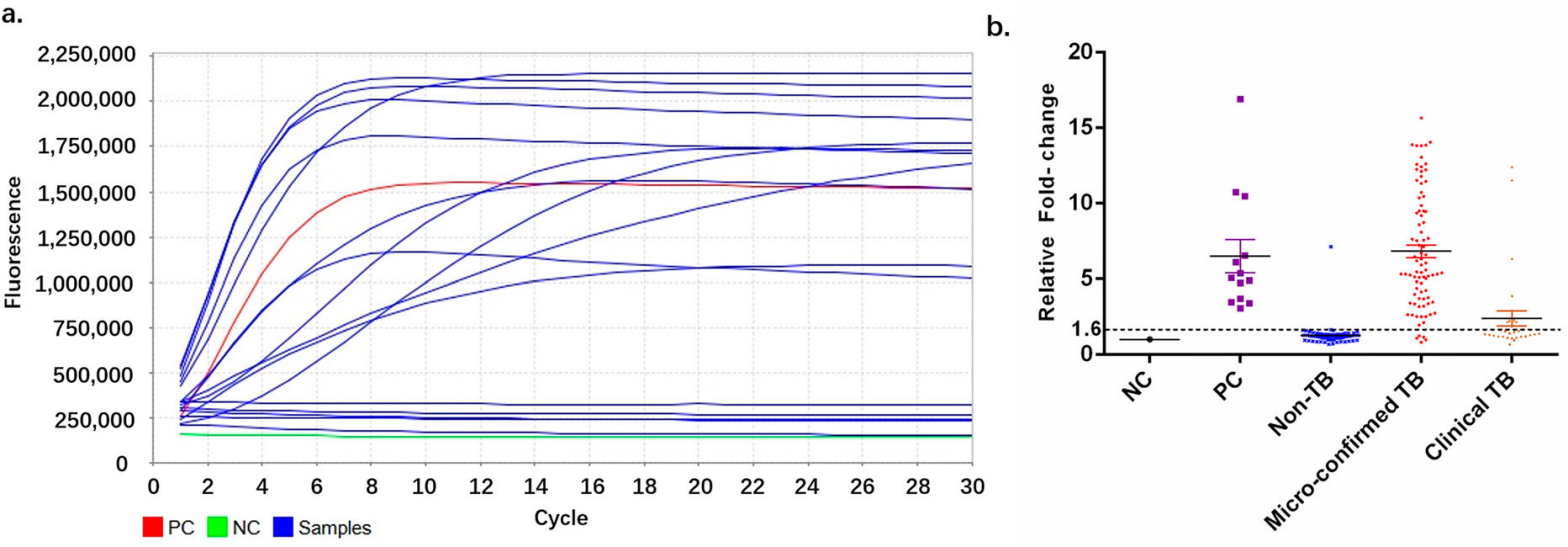
Cut-off value determination. (a) Typical fluorescent signal curve was presented by ABI7500 QPCR machine. (b) Fluorescent signals from each samples and positive controls were normalized by negative control of same batch. The folds changed were put together according indicated groups. “Non TB”, patients diagnosed with no active MTB infection. “Micro-confirmed TB”, patients diagnosed with active MTB infection by either culture or Xpert or both. “Clinical TB”, patients diagnosed with active MTB infection following Diagnostic criteria and principles of management of infectious MTB without evidence of either culture or GeneXpert.

All samples were analysed in 13 independent batches. For each testing, MTB and *M. kansasii* were served as positive (PC) and negative controls, respectively (Figure 4(a)). Signal from NC was used to normalize the signal of other samples including PC. All fold-changes of the PC were put together for cutoff determination. With the ROC curve analysis, samples yielded signals higher than 1.6 folds in the CRISPR assay were considered positive, while those lower than this cutoff were considered negative. By this cutoff, we identified 92 MTB-positive samples, and 87 non-MTB samples (Figure 4b).

We first looked at the performance of CRISPR-MTB in the subcohort of pulmonary tuberculosis cases (Table S2, Table S3). Out of the 51 cases that were clinically confirmed, 21 were positive by culture and 38 were detected by the Xpert assay, suggesting a sensitivity of 41% (21/51) vs 75% (38/51). This finding was in line with previous findings indicating the limited sensitivity of culture for TB detection [18]. In this group, CRISPR-MTB was able to pick up 46 out of 51 (90% sensitivity) pulmonary TB cases, including all 21 culture-positive cases and 37 of the Xpert-positive samples. This resulted in 90% (46/51) sensitivity for pulmonary TB, 100% (21/21) agreement in the culture-positive cases and 97%(37/38) agreement in the Xpert-positive cases, suggesting that CRISPR-MTB is a highly sensitive assay for pulmonary TB detection.

**Figure 5.**
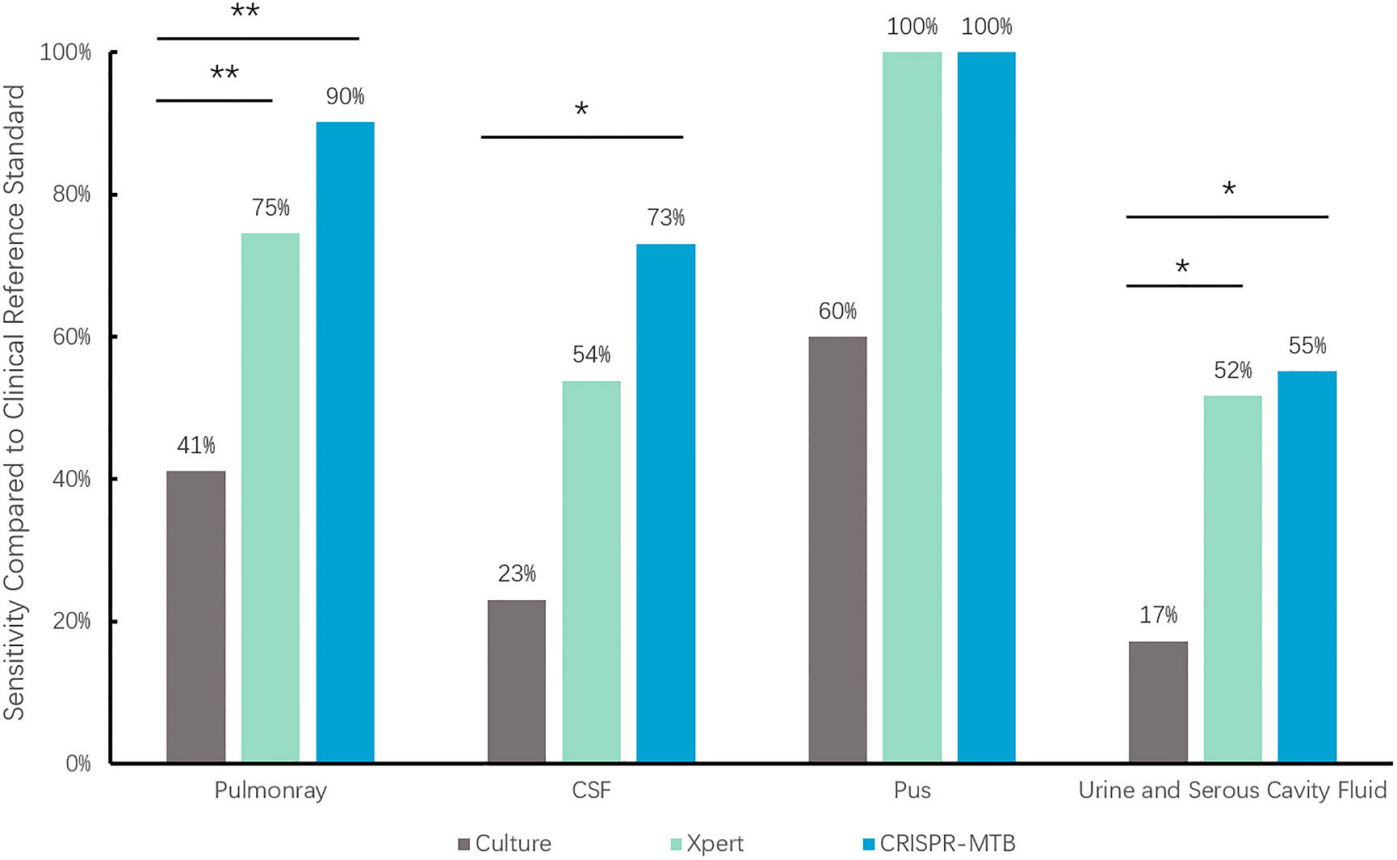
The sensitivity according to specimen type. * McNemar test, *P* < 0.01; ** McNemar test, *P* < 0.001

We next assessed the overall diagnostic performance of the CRISPR-MTB in the entire cohort of both pulmonary and extrapulmonary tuberculosis (Table S2, Table S3). Considering the substantial proportion of extrapulmonary cases in our cohort (56%, 65/116 TB patients) and the well-known limitation of the conventional method in diagnosing extrapulmonary MTB [19,20], we consider the final clinical diagnosis as the reference standard for comparison (Figure 4, Figure 5, Table S2). We found that CRISPR-MTB produced a sensitivity of 79% (91/116) in all active TB cases, which was significantly higher than Xpert (66%, 77/116, McNemar test, *P* = 0.004) and culture (33%, 38/116, McNemar test, *P* < 0.001). Remarkably, the CRISPR-MTB assay requires only 500μl of samples, which is much lower than the 1–6 ml demanded by the Xpert platform.

**Figure 6.**
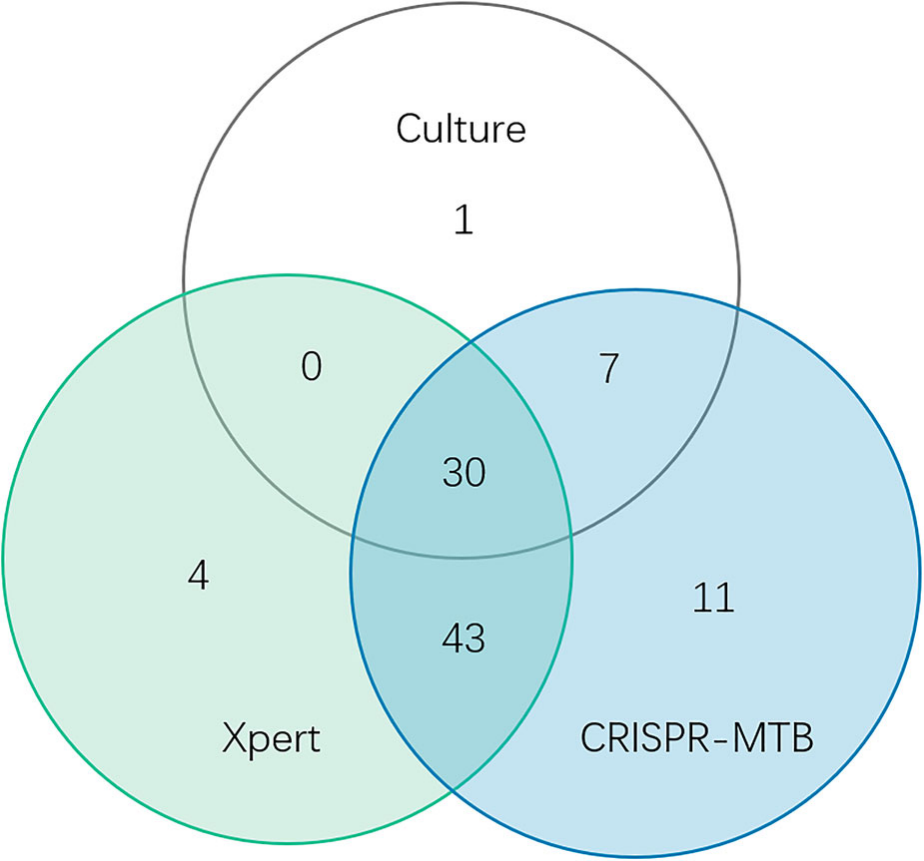
Venn diagram of overlap in TB diagnostic tests in micro-confirmed TB cases and clinically diagnose TB.

To further analyse the diagnostic performance, we compared these three methods in different sample types. We found that diagnostic performance varied among specimen types (Table S2, Table S3, Figure 5). As mentioned above, in 51 pulmonary TB cases, CRISPR-MTB reached sensitivity of 90%, which was also higher than Xpert (75%) and significantly superior to culture (41%, McNemar test, *P* < 0.001). In 26 CSF samples of clinical diagnosed tuberculous meningitis(TBM) patients, CRISPR-MTB had sensitivity of 73%, which was higher than Xpert (54%) and significantly higher than culture (23%, McNemar test, *P* = 0.001). For other extrapulmonary TB samples, CRISPR-MTB and Xpert showed comparable sensitivity and both identified 100% TB cases in pus samples and 55% in urine and serous cavity fluid samples. Both outperformed MTB culture significantly (100% vs 60% and 55% vs 17%, Figure 5). Also, it is worth noting that CRISPR-MTB was able to identify 11 clinically confirmed TB patients which would be otherwise missed by both Xpert and culture (Figure 6). These results suggest the overall higher sensitivity of CRISPR-MTB than both Xpert and culture for MTB detection.

The specificity of CRIPSR-MTB was examined in 63 non-TB cases, which consisted of patients of non-TB infections, malignancies and noninfectious inflammatory diseases. Importantly, CRISPR-MTB produced 1 false positive in all 63 non-TB cases and demonstrated a specificity of 98% (Table S2, Table S4).

**Figure 7.**
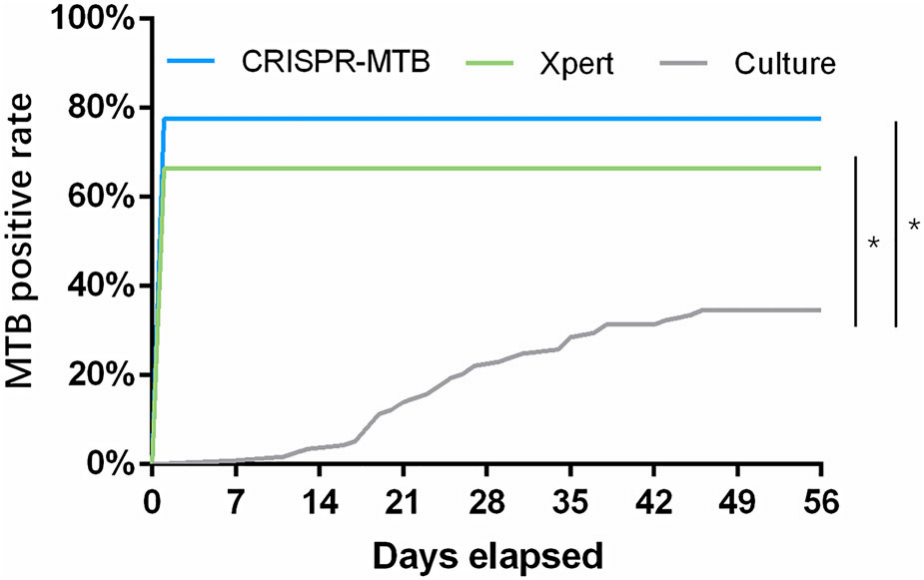
Kaplan–Meier curve of MTB positive rate by CRISPR-MTB, Xpert and culture. * Log-rank test, *P* < 0.0001.

When the turnaround time was compared, the CRISPR-MTB method evaluated in this study requires an average assay time of only 1.5 h, which includes 40 min of rapid DNA extraction, 30 min for DNA amplification by RPA and 20 min for Cas12a detection. This technique presents a significant advantage over culture and is even faster than the two-hour Xpert assay, allowing rapid, same-day MTB diagnosis in clinical practice (Figure 7).

## Discussion

DNA extraction remains a challenge for clinical MTB diagnosis. Traditional purification processes are usually complexed and time-consuming, and therefore not feasible for clinical application. Rapid extraction of MTB DNA was attempted by other groups using MTB strains or sputum as samples[19]. Unfortunately, these procedures did not perform well in our study due to the diversity of sample types. In this study, by optimizing the conditions of lysis and cell wall breaking, we established a rapid method that could adapt to a variety of sample types such as sputum, BALF, CSF and pus. This design takes advantage of both the polymerase-mediated DNA amplification and the Cas12a-mediated enzymatic signal amplification, and has been demonstrated to confer great sensitivity[6]. Moreover, as both reactions can occur at 37°C, it allows for an assay that does not demand a thermo-cycler. This protocol allows the entire testing procedure to be completed within as short as 1.5 h, and requires a small sample volume of 500 μl across all sample types assayed. As shown in Figure 2(b) in our manuscript, we found that the rapid extraction method produced comparable signal, suggesting a minimal inhibitory effect caused by sample processing. IS6110 has been well-recognized as a conserved genetic element in MTB and used as detection target in several commercial kits including Xpert MTB/RIF Ultra. Also, our study on 179 cases highlights targeting this element by CRISPR as a highly sensitive, promising assay for diagnosis for both pulmonary and extrapulmonary tuberculosis. Although rarely reported, we caution that these assays, including CRISPR-MTB, could miss MTB strains without any IS6110 copies. The false positives of the nucleic acid amplification tests method are mostly from the history of previous tuberculosis [20]. Although the patients with false-positive CRISPR-MTB result in our cohort denied a history of tuberculosis, it does not rule out the possibility of her previous concealed infection.

To the best of our knowledge, this is the first cohort study that aims to apply CRISPR for diagnosis of active MTB with direct clinical samples and to evaluate its diagnostic efficacy. We referred to the final clinical diagnosis rather than conventional methods as the reference standard. This is because that a large percentage (56%) of the cases in our cohort was extrapulmonary specimens suspected for tuberculosis, which is a weakness in conventional methods for MTB testing[21,22]. In addition, the potential advantages of newer tests could not be reflected if conventional methods were used as reference. Even though the clinical diagnosis lacks the evidence of culture or pathological results, the follow-up of more than 2 months greatly reduces the chance of misdiagnosis. Indeed, some NTMs are effective with anti-TB treatment, but in our cohort 90% of patients with clinically diagnosed pulmonary tuberculosis are positive for CRISPR-MTB, suggesting that this bias should be small if any. The lower positive rate of MTB culture for lung specimen is likely due to the paucibacillary nature of TB in our cohort. This is also suggested by our Xpert results showing that the vast majority of sputum samples are “low” or “very low” of MTB. We reasoned that TB patients referred to tertiary hospitals (as in this study) are more likely to be paucibacillary, as those with high MTB titre in sputum have a better chance to be already diagnosed in primary hospitals. Therefore, we believe that it is a suitable choice to use the clinical diagnosis as the reference standard when comparing across three diagnostic methods.

Among all types of specimens, the sensitivity of CRISPR-MTB was significantly higher than culture while not inferior to Xpert. In both pulmonary and extrapulmonary TB cases, CRISPR showed a satisfactory diagnostic efficacy compared to culture and Xpert in various specimen types including sputum, CSF, etc. In terms of diagnostic procedure, although the CRIPSR-MTB in its current format demands slightly more manual operation time than Xpert, its overall turnaround is shorter than Xpert, which makes it a promising alternative for rapid diagnosis of tuberculosis. It is very noteworthy that CRISPR-MTB requires much lower volume of specimens than Xpert. Specifically, for respiratory specimens and cerebrospinal fluid, the CRISPR-MTB requires only 500μl. In this study, the amount of urine and serous cavity fluid used for CRISPR-MTB was also less than one-quarter of the amount used in Xpert.

Our study highlights CRISPR as a highly sensitive, promising technology for *in vitro* diagnosis for both pulmonary and extrapulmonary tuberculosis. Given the low-complexity of the CRISPR-MTB method established in this study, it’s highly feasible to have the entire testing procedure completed at point-of-care by integrating it into a compact desktop machine for a sample-in-result-out assay[13]. Apart from diagnosis, drug resistance testing is also highly warranted for treating MTB patients. Genotypic analysis by molecular assays has been proven to offer great prediction value for resistance of MTB to drugs such as rifampicin and isoniazid. With the ultra sensitive nature of the CRISPR diagnostic technology, it holds the potential to simultaneously detect multiple sites related to drug response. An expanded CRISPR-MTB assay would be able to not only diagnose tuberculosis but also provide genetic insight into drug susceptibility for one- or even second-line drugs. Based on the proof-of-principle presented in this study for carrying out MTB testing directly from acquired clinical samples, rapid CRISPR detection of both pathogen and drug sensitivities would permit the precise approach to tuberculosis infection.

Although our study has shown excellent diagnostic value of using CRISPR for MTB detection in both pulmonary and extra-pulmonary tuberculosis, future multi-centre prospective study would provide deeper understanding on its potential for clinical diagnosis.

## Conclusion

The current study presented for the first time a CRISPR-based method for active tuberculosis diagnosis with ultra, near single-copy analytical sensitivity. The assay was further evaluated in a cohort study consisting of both pulmonary and extra-pulmonary tuberculosis cases. The CRISPR-MTB test exhibited an improved diagnostic performance over culture and Xpert not only in higher sensitivity, but also in lower sample input and shorter turnaround time.

## Supplementary Material

Supplemental Material
