# Supplementary material for: CRISPR-based rapid and ultra-sensitive diagnostic test for Mycobacterium tuberculosis
Source: Emerg Microbes Infect. 2019 Sep 15;8(1):1361–9. doi: 10.1080/22221751.2019.1664939 (PMC6758691; doi:10.1080/22221751.2019.1664939)
Supplement: Supplemental Material [file TEMI_A_1664939_SM9205.zip › TEMI_2019_0276.R2_Supplemental_materials_final.docx]

**Supplemental materials**

**Sputum decontamination**

Clinical specimens were decontaminated using the N-acetyl-L-cysteine sodium hydroxide method (NALC-NaOH). After centrifugation, the pellet was resuspended in 1 to 1.5 ml of sterile phosphate buffer (pH 6.8). This suspension was used for inoculation of culture media. A smear of the processed sample was prepared and examined for the presence of AFB.

**MTBC culture**

Liquid culture media based on fluorometric detection of growth. Mycobacteria Growth Indicator Tube (MGIT) tubes were inoculated with 0.5 ml of the processed specimen. The tubes were incubated in the MGIT 960 instrument at 37°C.

Solid culture media was inoculated with 0.25 ml suspension processed for each specimen and incubated at 37°C. For tubes identified as positive, a smear of a sample from the tube was prepared for examination for AFB. All smears were stained by the Kinyoun method and examined with a light microscope. MTB strains isolated from culture were identified using the MGIT TBc ID method (MPT 64: Becton Dickinson, Sparks, Maryland, USA).

**Figure S1** The proportion of positive samples detected by CRISPR-MTB at different MTB-DNA concentrations (n=10).

**Figure S2** LoD evaluation in quantified CFU. **** P<0.0001

**Figure S3** The proportion of positive samples detected by CRISPR-MTB in the purified DNA of multiple different organisms (n=3).

**Table S1.** Baseline characteristics of the study population.

|  |  | Micro-confirmed TB (n=85) | Clinical diagnosed TB (n=31) | Non-TB  (n=63) |
| --- | --- | --- | --- | --- |
| Age (Mean)^#^, years (range) | | 45.47(18-77) | 53.67(23-85) | 55.19(20-87) |
| Sex-Male*, no.(%) | | 47(55.3%) | 13(41.9%) | 45(71.4%) |
| Laboratory parameters | | | |  |
|  | Hb (Mean), g/L | 117.4 | 111.2 | 116.2 |
|  | WBC (Mean), 10^9^/L | 6.9 | 6.7 | 8.0 |
|  | T.SPOT *TB* (+)**,no.(%) | 48(56.5%) | 21(67.7%) | 13(20.6%) |
| Pulmonary samples, no. | | | |  |
|  | Sputum | 11 | 0 | 5 |
|  | BALF | 30 | 10 | 20 |
| Extrapulmonary samples, no. | | | |  |
|  | CSF | 18 | 8 | 14 |
|  | Pleural fluid | 4 | 4 | 10 |
|  | Ascites | 3 | 5 | 6 |
|  | Pus | 10 | 0 | 4 |
|  | Pericardial effusion | 1 | 1 | 0 |
|  | Urine | 8 | 3 | 3 |
|  | Synovial fluid | 0 | 0 | 1 |
| Symptom | | | |  |
|  | Headache | 17 | 6 | 11 |
|  | Fever | 47 | 23 | 32 |
|  | Confusion | 17 | 4 | 5 |
|  | emaciation | 21 | 8 | 14 |
|  | Cough | 42 | 13 | 30 |
|  | haemoptysis | 8 | 0 | 3 |
|  | abdominal distention | 6 | 2 | 9 |
|  | abdominal pain | 2 | 1 | 2 |
|  | Fatigue | 9 | 7 | 10 |
|  | Multiple enlarged lymph nodes | 9 | 4 | 4 |
|  | low back pain | 5 | 0 | 5 |
|  | chest pain | 4 | 3 | 5 |
|  | Urgency | 6 | 0 | 4 |
|  | arthralgia | 1 | 0 | 2 |
|  | scrotal hydrocele | 1 | 1 | 0 |
|  | paraplegia | 3 | 0 | 0 |
|  | cutaneous dropsy | 1 | 0 | 0 |
|  | arm pain | 1 | 0 | 0 |

WBC: white blood cell count, CSF: cerebrospinal fluid, BALF: bronchoalveolar lavage fluid, ATT: anti-TB treatment.

^#^ P=0.0084, T test; *P=0.0114, **P<0.0001, Fisher’s exact test.

**Table S2.** Diagnostic performance of MTB for CRISPR-MTB, Xpert and culture compared with the clinical reference standard.

|  |  | Sensitivity  (95%CI; n/N) | Specificity  (n/N) |
| --- | --- | --- | --- |
| All samples | | | |
| * | Culture | 33% (0.24 to 0.42; 38/116) | 100% (63/63) |
| * | Xpert | 66% (0.57 to 0.75; 77/116) | 100% (63/63) |
| # |  |  |  |
|  | CRISPR-MTB | 79% (0.70 to 0.86; 91/116) | 98% (62/63) |
| Pulmonary samples | | | |
| * | Culture | 41% (0.28 to 0.56; 21/51) | 100% (25/25) |
| * | Xpert | 75% (0.60 to 0.86; 38/51) | 100% (25/25) |
|  | CRISPR-MTB | 90% (0.79 to 0.97; 46/51) | 96% (24/25) |
| Extrapulmonry samples | | | |
| * | Culture | 26% (0.16 to 0.39; 17/65) | 100%(38/38) |
| * | Xpert | 60% (0.47-0.72; 39/65) | 100%(38/38) |
|  | CRISPR-MTB | 69% (0.57-0.80; 45/65) | 100%(38/38) |

* McNemar test, P<0.001；# McNemar test, P=0.004

**Table S3.** Correlation of CRISPR-MTB assay versus culture and Xpert.

|  |  |  | Culture | | Xpert | |
| --- | --- | --- | --- | --- | --- | --- |
|  |  |  | + | - | + | - |
| Pulmonary | CRISPR-MTB | + | 21 | 25 | 37 | 9 |
|  |  | - | 0 | 5 | 1 | 4 |
| CSF | CRISPR-MTB | + | 5 | 14 | 13 | 6 |
|  |  | - | 1 | 6 | 1 | 6 |
| Pus | CRISPR-MTB | + | 6 | 4 | 10 | 0 |
|  |  | - | 0 | 0 | 0 | 0 |
| Urine and serous cavity fluid | CRISPR-MTB | + | 5 | 11 | 13 | 3 |
|  |  | - | 0 | 13 | 2 | 11 |

**Table S4**. Characteristics of enrolled cases.

| **Case No.** | **Symptoms** | **Sample type** | **MTB Culture** | **Xpert** | **CRISPR-MTB** | **Folds-change** | **TB diagnosis** | **Clinical diagnosis** |
| --- | --- | --- | --- | --- | --- | --- | --- | --- |
| 1 | fever, confusion | CSF | (-) | (-) | (-) | 1.4 | Clinical TB | CNS TB |
| 2 | headache, fever, confusion | CSF | (-) | (-) | (-) | 1.6 | Clinical TB | CNS TB |
| 3 | fever emaciation | CSF | (-) | (-) | (+) | 2.2 | Clinical TB | CNS TB |
| 4 | headache, fever, confusion, cough, abdominal pain | CSF | (-) | (-) | (-) | 1.3 | Clinical TB | CNS TB |
| 5 | headache, fever emaciation | CSF | (-) | (-) | (+) | 3.9 | Clinical TB | CNS TB |
| 6 | headache, fever | CSF | (-) | (-) | (-) | 1.4 | Clinical TB | CNS TB |
| 7 | headache, fever | CSF | (-) | (-) | (+) | 2.2 | Clinical TB | CNS TB |
| 8 | fever, confusion, | CSF | (-) | (-) | (-) | 1.0 | Clinical TB | CNS TB |
| 9 | fever emaciation, fatigue scrotal hydrocele | BALF | (-) | (-) | (-) | 1.3 | Clinical TB | Pulmonary TB |
| 10 | fever, cough, | BALF | (-) | (-) | (-) | 1.6 | Clinical TB | Pulmonary TB |
| 11 | fever, cough, | BALF | (-) | (-) | (-) | 1.3 | Clinical TB | Pulmonary TB |
| 12 | cough | BALF | (-) | (-) | (+) | 2.3 | Clinical TB | Pulmonary TB |
| 13 | cough | BALF | (-) | (-) | (+) | 2.9 | Clinical TB | Pulmonary TB |
| 14 | cough，fatigue | BALF | (-) | (-) | (-) | 0.7 | Clinical TB | Pulmonary TB |
| 15 | chest pain | BALF | (-) | (-) | (+) | 11.5 | Clinical TB | Pulmonary TB |
| 16 | fever, emaciation, cough, fatigue | BALF | (-) | (-) | (+) | 6.3 | Clinical TB | Pulmonary TB |
| 17 | fever, emaciation, cough | BALF | (-) | (-) | (+) | 12.4 | Clinical TB | Pulmonary TB |
| 18 | fever, emaciation, cough | BALF | (-) | (-) | (+) | 1.9 | Clinical TB | Pulmonary TB |
| 19 | chest pain | Pericardial effusion | (-) | (-) | (-) | 1.1 | Clinical TB | TB pericarditis |
| 20 | emaciation, abdominal distention fatigue | Ascites | (-) | (-) | (-) | 1.2 | Clinical TB | TB Peritonitis |
| 21 | abdominal distention, fatigue | Ascites | (-) | (-) | (-) | 1.5 | Clinical TB | TB Peritonitis |
| 22 | fever, cough | Ascites | (-) | (-) | (-) | 1.3 | Clinical TB | TB Peritonitis |
| 23 | fever， multiple lymphode enlargement | Ascites | (-) | (-) | (-) | 1.2 | Clinical TB | TB Peritonitis |
| 24 | fever， multiple enlarged lymph nodes | Ascites | (-) | (-) | (-) | 1.1 | Clinical TB | TB Peritonitis |
| 25 | fever, fatigue, multiple enlarged lymph nodes | Pleural fluid | (-) | (-) | (+) | 1.7 | Clinical TB | TB Pleurisy |
| 26 | chest pain | Pleural fluid | (-) | (-) | (-) | 1.5 | Clinical TB | TB Pleurisy |
| 27 | fever emaciation, cough | Pleural fluid | (-) | (-) | (-) | 1.2 | Clinical TB | TB Pleurisy |
| 28 | fever, cough, | Pleural fluid | (-) | (-) | (-) | 1.2 | Clinical TB | TB Pleurisy |
| 29 | fever, cough, multiple enlarged lymph nodes | Urine | (-) | (-) | (-) | 1.3 | Clinical TB | Urinary tract TB |
| 30 | fever, fatigue, | Urine | (-) | (-) | (-) | 1.1 | Clinical TB | Urinary tract TB |
| 31 | headache, fever | Urine | (-) | (-) | (+) | 2.2 | Clinical TB | Urinary tract TB |
| 32 | headache, fever, cough, haemoptysis | CSF | (+) | (-) | (+) | 2.1 | Micro-confirmed TB | CNS TB |
| 33 | headache, fever, confusion | CSF | (-) | (+) | (+) | 5.1 | Micro-confirmed TB | CNS TB |
| 34 | fever emaciation | CSF | (+) | (+) | (+) | 2.5 | Micro-confirmed TB | CNS TB |
| 35 | headache confusion, emaciation, cough abdominal distention, abdominal pain | CSF | (+) | (+) | (+) | 3.5 | Micro-confirmed TB | CNS TB |
| 36 | headache, fever | CSF | (+) | (-) | (+) | 2.5 | Micro-confirmed TB | CNS TB |
| 37 | headache, fever | CSF | (+) | (-) | (-) | 1.0 | Micro-confirmed TB | CNS TB |
| 38 | fever, confusion | CSF | (-) | (+) | (+) | 2.0 | Micro-confirmed TB | CNS TB |
| 39 | headache, fever, confusion | CSF | (-) | (+) | (-) | 1.2 | Micro-confirmed TB | CNS TB |
| 40 | headache, fever, confusion | CSF | (-) | (+) | (+) | 6.3 | Micro-confirmed TB | CNS TB |
| 41 | headache, low back pain | CSF | (-) | (+) | (+) | 7.6 | Micro-confirmed TB | CNS TB |
| 42 | headache, fever, confusion, emaciation, cough, | CSF | (-) | (+) | (+) | 7.1 | Micro-confirmed TB | CNS TB |
| 43 | headache, fever, confusion | CSF | (-) | (+) | (+) | 6.2 | Micro-confirmed TB | CNS TB |
| 44 | headache, fever, confusion, emaciation | CSF | (-) | (+) | (+) | 9.2 | Micro-confirmed TB | CNS TB |
| 45 | headache, fever, confusion fatigue, | CSF | (-) | (+) | (+) | 9.4 | Micro-confirmed TB | CNS TB |
| 46 | fever, confusion | CSF | (-) | (+) | (+) | 4.4 | Micro-confirmed TB | CNS TB |
| 47 | headache, | CSF | (-) | (+) | (+) | 9.8 | Micro-confirmed TB | CNS TB |
| 48 | headache, fever | CSF | (-) | (+) | (+) | 4.0 | Micro-confirmed TB | CNS TB |
| 49 | headache, fever, confusion | CSF | (+) | (-) | (+) | 2.7 | Micro-confirmed TB | CNS TB |
| 50 | fever, cough, multiple enlarged lymph nodes | BALF | (-) | (+) | (+) | 12.6 | Micro-confirmed TB | Pulmonary TB |
| 51 | fever, emaciation, cough | BALF | (+) | (+) | (+) | 11.3 | Micro-confirmed TB | Pulmonary TB |
| 52 | emaciation | BALF | (-) | (+) | (+) | 13.8 | Micro-confirmed TB | Pulmonary TB |
| 53 | fever, emaciation, cough, fatigue | BALF | (+) | (+) | (+) | 9.5 | Micro-confirmed TB | Pulmonary TB |
| 54 | emaciation, cough | BALF | (+) | (-) | (+) | 13.9 | Micro-confirmed TB | Pulmonary TB |
| 55 | multiple enlarged lymph nodes | BALF | (-) | (+) | (+) | 12.2 | Micro-confirmed TB | Pulmonary TB |
| 56 | emaciation, cough | BALF | (+) | (+) | (+) | 13.8 | Micro-confirmed TB | Pulmonary TB |
| 57 | cough, fatigue | BALF | (+) | (-) | (+) | 11.4 | Micro-confirmed TB | Pulmonary TB |
| 58 | fever, cough | BALF | (+) | (+) | (+) | 8.7 | Micro-confirmed TB | Pulmonary TB |
| 59 | cough, chest pain | BALF | (+) | (+) | (+) | 13.9 | Micro-confirmed TB | Pulmonary TB |
| 60 | chest pain | BALF | (-) | (+) | (+) | 12.1 | Micro-confirmed TB | Pulmonary TB |
| 61 | fever, emaciation, cough | BALF | (+) | (+) | (+) | 9.5 | Micro-confirmed TB | Pulmonary TB |
| 62 | emaciation, cough | BALF | (-) | (+) | (+) | 12.4 | Micro-confirmed TB | Pulmonary TB |
| 63 | cough | BALF | (-) | (+) | (+) | 11.8 | Micro-confirmed TB | Pulmonary TB |
| 64 | fever, emaciation, cough | BALF | (-) | (+) | (+) | 4.7 | Micro-confirmed TB | Pulmonary TB |
| 65 | cough | BALF | (+) | (+) | (+) | 5.5 | Micro-confirmed TB | Pulmonary TB |
| 66 | fever, cough, | BALF | (-) | (+) | (+) | 5.1 | Micro-confirmed TB | Pulmonary TB |
| 67 | cough | Sputum | (+) | (+) | (+) | 3.4 | Micro-confirmed TB | Pulmonary TB |
| 68 | fever emaciation, cough | BALF | (+) | (+) | (+) | 5.1 | Micro-confirmed TB | Pulmonary TB |
| 69 | fever emaciation, cough, | Sputum | (-) | (+) | (+) | 5.0 | Micro-confirmed TB | Pulmonary TB |
| 70 | fever, confusion cough, haemoptysis | BALF | (-) | (+) | (+) | 6.5 | Micro-confirmed TB | Pulmonary TB |
| 71 | fever chest pain | BALF | (+) | (-) | (+) | 2.6 | Micro-confirmed TB | Pulmonary TB |
| 72 | fever, cough | Sputum | (+) | (+) | (+) | 5.3 | Micro-confirmed TB | Pulmonary TB |
| 73 | paraplegia | BALF | (-) | (+) | (+) | 4.2 | Micro-confirmed TB | Pulmonary TB |
| 74 | fever, cough, | BALF | (-) | (+) | (+) | 5.2 | Micro-confirmed TB | Pulmonary TB |
| 75 | cough, cutaneous dropsy | Sputum | (-) | (+) | (+) | 5.4 | Micro-confirmed TB | Pulmonary TB |
| 76 | headache, fever, confusion, emaciation, cough | Sputum | (-) | (+) | (+) | 3.2 | Micro-confirmed TB | Pulmonary TB |
| 77 | emaciation, cough, | Sputum | (+) | (+) | (+) | 7.2 | Micro-confirmed TB | Pulmonary TB |
| 78 | confusion, cough | Sputum | (-) | (+) | (-) | 0.8 | Micro-confirmed TB | Pulmonary TB |
| 79 | cough | BALF | (-) | (+) | (+) | 12.2 | Micro-confirmed TB | Pulmonary TB |
| 80 | cough | Sputum | (+) | (+) | (+) | 14.1 | Micro-confirmed TB | Pulmonary TB |
| 81 | cough, haemoptysis | BALF | (-) | (+) | (+) | 13.0 | Micro-confirmed TB | Pulmonary TB |
| 82 | cough | BALF | (+) | (+) | (+) | 11.5 | Micro-confirmed TB | Pulmonary TB |
| 83 | cough | BALF | (+) | (+) | (+) | 6.6 | Micro-confirmed TB | Pulmonary TB |
| 84 | cough, haemoptysis | Sputum | (+) | (+) | (+) | 10.7 | Micro-confirmed TB | Pulmonary TB |
| 85 | cough | BALF | (-) | (+) | (+) | 15.6 | Micro-confirmed TB | Pulmonary TB |
| 86 | cough | BALF | (+) | (+) | (+) | 12.6 | Micro-confirmed TB | Pulmonary TB |
| 87 | haemoptysis | Sputum | (+) | (+) | (+) | 10.5 | Micro-confirmed TB | Pulmonary TB |
| 88 | fever, cough | Sputum | (-) | (+) | (+) | 10.4 | Micro-confirmed TB | Pulmonary TB |
| 89 | fever, confusion, emaciation, cough | BALF | (-) | (+) | (+) | 3.2 | Micro-confirmed TB | Pulmonary TB |
| 90 | cough, haemoptysis | BALF | (+) | (+) | (+) | 3.8 | Micro-confirmed TB | Pulmonary TB |
| 91 | fever, cough, arthralgia | Pus | (+) | (+) | (+) | 5.6 | Micro-confirmed TB | Soft tissue TB |
| 92 | fever | Pus | (-) | (+) | (+) | 4.8 | Micro-confirmed TB | Soft tissue TB |
| 93 | fever, multiple enlarged lymph nodes | Pus | (+) | (+) | (+) | 5.2 | Micro-confirmed TB | Soft tissue TB |
| 94 | headache, low back pain | Pus | (+) | (+) | (+) | 5.9 | Micro-confirmed TB | Soft tissue TB |
| 95 | low back pain | Pus | (+) | (+) | (+) | 3.4 | Micro-confirmed TB | Soft tissue TB |
| 96 | multiple enlarged lymph nodes | Pus | (-) | (+) | (+) | 3.7 | Micro-confirmed TB | Soft tissue TB |
| 97 | cough, multiple enlarged lymph nodes | Pus | (-) | (+) | (+) | 2.6 | Micro-confirmed TB | Soft tissue TB |
| 98 | fever, multiple enlarged lymph nodes | Pus | (-) | (+) | (+) | 7.7 | Micro-confirmed TB | Soft tissue TB |
| 99 | fever, arm pain | Pus | (+) | (+) | (+) | 4.2 | Micro-confirmed TB | Soft tissue TB |
| 100 | fever, multiple enlarged lymph nodes | Pus | (+) | (+) | (+) | 5.3 | Micro-confirmed TB | Soft tissue TB |
| 101 | , emaciation, abdominal distention, chest pain | Pericardial effusion | (-) | (+) | (+) | 6.7 | Micro-confirmed TB | TB pericarditis |
| 102 | abdominal distention, fatigue | Ascites | (-) | (+) | (+) | 5.3 | Micro-confirmed TB | TB Peritonitis |
| 103 | fever, emaciation, abdominal distention, fatigue, | Ascites | (+) | (+) | (+) | 5.2 | Micro-confirmed TB | TB Peritonitis |
| 104 | fever, abdominal distention, fatigue, paraplegia | Ascites | (+) | (-) | (+) | 3.7 | Micro-confirmed TB | TB Peritonitis |
| 105 | fever, fatigue, multiple enlarged lymph nodes, paraplegia | Pleural fluid | (-) | (+) | (-) | 1.2 | Micro-confirmed TB | TB Pleurisy |
| 106 | fever, emaciation, fatigue, multiple enlarged lymph nodes | Pleural fluid | (-) | (+) | (-) | 1.1 | Micro-confirmed TB | TB Pleurisy |
| 107 | fever, confusion, emaciation, cough, haemoptysis, fatigue | Pleural fluid | (+) | (+) | (+) | 8.1 | Micro-confirmed TB | TB Pleurisy |
| 108 | fever emaciation, cough | Pleural fluid | (-) | (+) | (+) | 2.5 | Micro-confirmed TB | TB Pleurisy |
| 109 | fever, scrotal hydrocele | Urine | (-) | (+) | (+) | 7.6 | Micro-confirmed TB | Urinary tract TB |
| 110 | fever, urgency | Urine | (+) | (+) | (+) | 7.5 | Micro-confirmed TB | Urinary tract TB |
| 111 | fever, abdominal distention, abdominal pain, urgency | Urine | (-) | (+) | (+) | 8.6 | Micro-confirmed TB | Urinary tract TB |
| 112 | low back pain, urgency | Urine | (+) | (+) | (+) | 3.4 | Micro-confirmed TB | Urinary tract TB |
| 113 | fever, low back pain, urgency | Urine | (-) | (+) | (+) | 5.4 | Micro-confirmed TB | Urinary tract TB |
| 114 | urgency | Urine | (-) | (+) | (+) | 2.6 | Micro-confirmed TB | Urinary tract TB |
| 115 | urgency | Urine | (-) | (+) | (+) | 9.4 | Micro-confirmed TB | Urinary tract TB |
| 116 | confusion | Urine | (-) | (+) | (+) | 6.0 | Micro-confirmed TB | Urinary tract TB |
| 117 | headache, fever, cough, fatigue, arthralgia | CSF | (-) | (-) | (-) | 1.0 | Non-TB | Bacteria infection (Streptococcus Infectious endocarditis) |
| 118 | low back pain, urgency | Urine | (-) | (-) | (-) | 1.3 | Non-TB | Bacterial infeciton (Enterococcus faecalis urinary tract infection) |
| 119 | fever, urgency | Urine | (-) | (-) | (-) | 1.0 | Non-TB | Bacterial infection (Escherichia coli urinary tract infection) |
| 120 | emaciation, urgency | Urine | (-) | (-) | (-) | 1.4 | Non-TB | Bacterial infection (Escherichia coli urinary tract infection) |
| 121 | cough | BALF | (-) | (-) | (-) | 1.4 | Non-TB | Bacterial infection (Bacterial pneumonia) |
| 122 | headache, fever, cough | BALF | (-) | (-) | (-) | 1.4 | Non-TB | Bacterial infection (Bacterial pneumonia) |
| 123 | cough | BALF | (-) | (-) | (-) | 1.6 | Non-TB | Bacterial infection (Bacterial pneumonia) |
| 124 | cough | BALF | (-) | (-) | (-) | 1.0 | Non-TB | Bacterial infection (Bacterial pneumonia) |
| 125 | cough | BALF | (-) | (-) | (-) | 0.9 | Non-TB | Bacterial infection (Bacterial pneumonia) |
| 126 | cough | BALF | (-) | (-) | (-) | 1.3 | Non-TB | Bacterial infection (Bacterial pneumonia) |
| 127 | fever, emaciation, cough, fatigue | BALF | (-) | (-) | (-) | 1.3 | Non-TB | Bacterial infection (Bacterial pneumonia) |
| 128 | fever, emaciation, low back pain | Pus | (-) | (-) | (-) | 0.8 | Non-TB | Bacterial infection (Bacteroides fragilis abcess) |
| 129 | arthralgia | Synovial fluid | (-) | (-) | (-) | 1.2 | Non-TB | Bacterial infection (Infectious arthritis) |
| 130 | fever, cough | BALF | (-) | (-) | (-) | 1.6 | Non-TB | Bacterial infection (Klebsiella pneumoniae pneumonia) |
| 131 | fever, cough, multiple enlarged lymph nodes | Pleural fluid | (-) | (-) | (-) | 0.9 | Non-TB | Bacterial infection (Legionella pneumonia) |
| 132 | fever | Pus | (-) | (-) | (-) | 0.8 | Non-TB | Bacterial infection (M. colombiense liver abcess) |
| 133 | chest pain | BALF | (-) | (-) | (-) | 1.6 | Non-TB | Bacterial infection (Mycobacterium intracellulare pneumonia) |
| 134 | headache | CSF | (-) | (-) | (-) | 1.4 | Non-TB | Bacterial infection (Neurosyphilis) |
| 135 | fever, fatigue, | Pleural fluid | (-) | (-) | (-) | 0.9 | Non-TB | Bacterial infection (NTM pneumonia) |
| 136 | fever, cough | BALF | (-) | (-) | (-) | 1.5 | Non-TB | Bacterial infection (Pseudomonas aeruginosa pneumonia) |
| 137 | fever, confusion, emaciation, fatigue, urgency | CSF | (-) | (-) | (-) | 1.1 | Non-TB | Bacterial infection (Scrub typhus) |
| 138 | fever, chest pain | Pus | (-) | (-) | (-) | 0.8 | Non-TB | Bacterial infection (Streptococcus pneumoia) |
| 139 | cough | Sputum | (-) | (-) | (-) | 0.9 | Non-TB | Fungal infection (Candida pneumonia) |
| 140 | headache, fever | CSF | (-) | (-) | (-) | 1.1 | Non-TB | Fungal infection (Cryptococcal meningitis) |
| 141 | fever, cough | Sputum | (-) | (-) | (-) | 0.9 | Non-TB | Fungal infection (Cryptococcal pneumoina) |
| 142 | fever, multiple enlarged lymph nodes | Pus | (-) | (-) | (-) | 0.7 | Non-TB | Fungal infection (Penicillium marneffei Abecess) |
| 143 | fever, cough | BALF | (-) | (-) | (-) | 1.4 | Non-TB | Fungal infection (Pulmonary aspergillosis) |
| 144 | fever emaciation, cough, haemoptysis | BALF | (-) | (-) | (-) | 1.1 | Non-TB | Fungal infection (Pulmonary aspergillosis) |
| 145 | confusion, | CSF | (-) | (-) | (-) | 1.4 | Non-TB | Malignancies (Brain metastases from non-small cell lung cancer) |
| 146 | headache, fever | CSF | (-) | (-) | (-) | 0.9 | Non-TB | Malignancies (Brain metastases) |
| 147 | fever | CSF | (-) | (-) | (-) | 1.2 | Non-TB | Malignancies (CNS lymphoma) |
| 148 | emaciation | CSF | (-) | (-) | (-) | 1.2 | Non-TB | Malignancies (CNS tumor) |
| 149 | headache, fever, cough, | BALF | (-) | (-) | (-) | 1.0 | Non-TB | Malignancies (MALT lymphoma) |
| 150 | fever emaciation, fatigue, multiple enlarged lymph nodes | Ascites | (-) | (-) | (-) | 1.1 | Non-TB | Malignancies (Mesothelioma) |
| 151 | emaciation, abdominal distention, abdominal pain | Ascites | (-) | (-) | (-) | 1.3 | Non-TB | Malignancies (Metastases) |
| 152 | headache, fever, cough, chest pain | Pleural fluid | (-) | (-) | (-) | 1.2 | Non-TB | Malignancies (Non Hodgin's lymphoma) |
| 153 | fever, confusion | CSF | (-) | (-) | (-) | 1.1 | Non-TB | Malignancies (Non Hodgin's lymphoma) |
| 154 | fever emaciation, abdominal distention, abdominal pain multiple enlarged lymph nodes | Ascites | (-) | (-) | (-) | 1.1 | Non-TB | Malignancies (Non Hodgin's lymphoma) |
| 155 | fever, cough | Pleural fluid | (-) | (-) | (-) | 1.3 | Non-TB | Malignancies (Non Hodgin's lymphoma) |
| 156 | emaciation, low back pain | Pleural fluid | (-) | (-) | (-) | 1.3 | Non-TB | Malignancies (Non-small cell lung cancer) |
| 157 | low back pain | Pleural fluid | (-) | (-) | (-) | 1.3 | Non-TB | Malignancies (Non-small cell lung cancer) |
| 158 | cough, fatigue, chest pain | BALF | (-) | (-) | (-) | 1.4 | Non-TB | Malignancies (Non-small cell lung cancer) |
| 159 | emaciation, cough, fatigue | BALF | (-) | (-) | (-) | 1.3 | Non-TB | Malignancies (Non-small cell lung cancer) |
| 160 | cough | BALF | (-) | (-) | (+) | 7.1 | Non-TB | Malignancies (Non-small cell lung cancer) |
| 161 | headache, fever | CSF | (-) | (-) | (-) | 1.3 | Non-TB | Malignancies (Schwannoma) |
| 162 | fever, cough, chest pain | BALF | (-) | (-) | (-) | 1.5 | Non-TB | Miscellaneous causes (Acute exacerbations of chronic obstructive pulmonary disease) |
| 163 | cough, haemoptysis | BALF | (-) | (-) | (-) | 1.2 | Non-TB | Miscellaneous causes (Bronchiectasis) |
| 164 | abdominal distention | Ascites | (-) | (-) | (-) | 1.0 | Non-TB | Miscellaneous causes (Decompensentated liver cirrhosis) |
| 165 | cough, haemoptysis, abdominal distention | Pleural fluid | (-) | (-) | (-) | 1.1 | Non-TB | Miscellaneous causes (Decompensentated liver cirrhosis) |
| 166 | abdominal distention, fatigue | Pleural fluid | (-) | (-) | (-) | 1.1 | Non-TB | Miscellaneous causes (Decompensentated liver cirrhosis) |
| 167 | emaciation, abdominal distention, fatigue | Pleural fluid | (-) | (-) | (-) | 1.3 | Non-TB | Miscellaneous causes (Decompensentated liver cirrhosis) |
| 168 | cough, low back pain | Pleural fluid | (-) | (-) | (-) | 1.2 | Non-TB | Miscellaneous causes (Hypoproteinemia) |
| 169 | headache, fever | CSF | (-) | (-) | (-) | 1.3 | Non-TB | Miscellaneous causes (Moyamoya disease) |
| 170 | emaciation, cough, fatigue | Sputum | (-) | (-) | (-) | 0.8 | Non-TB | Miscellaneous causes (Organizing pneumonia, COP) |
| 171 | abdominal distention | Ascites | (-) | (-) | (-) | 1.0 | Non-TB | Miscellaneous causes (Primary peritonitis) |
| 172 | cough | BALF | (-) | (-) | (-) | 1.4 | Non-TB | Miscellaneous causes(chronic obstructive pulmonary disease) |
| 173 | headache, fever, confusion | CSF | (-) | (-) | (-) | 1.1 | Non-TB | Noninfectious inflammatory disease (acute disseminated encephalomyelitis) |
| 174 | fever | CSF | (-) | (-) | (-) | 1.3 | Non-TB | Noninfectious inflammatory disease (Acute myelitis) |
| 175 | fever , emaciation, cough | Sputum | (-) | (-) | (-) | 0.8 | Non-TB | Noninfectious inflammatory disease (ANCA Vasculitis) |
| 176 | cough | BALF | (-) | (-) | (-) | 1.6 | Non-TB | Noninfectious inflammatory disease (asthma) |
| 177 | emaciation, abdominal distention | Ascites | (-) | (-) | (-) | 1.2 | Non-TB | Noninfectious inflammatory disease (Overlapping syndrome) |
| 178 | fever, cough | Sputum | (-) | (-) | (-) | 0.9 | Non-TB | Viral infection (Influenza A pneumonia) |
| 179 | headache, fever, confusion | CSF | (-) | (-) | (-) | 0.7 | Non-TB | Viral infection (Viral encephalitis) |

(-) Negative for MTB detection; (+) Positive for MTB detection. CSF: cerebrospinal fluid, BALF: bronchoalveolar lavage fluid.
