# Supplementary figures and images for: CRISPR-based rapid and ultra-sensitive diagnostic test for Mycobacterium tuberculosis
Source: Emerg Microbes Infect. 2019 Sep 15;8(1):1361–9. doi: 10.1080/22221751.2019.1664939 (PMC6758691; doi:10.1080/22221751.2019.1664939)

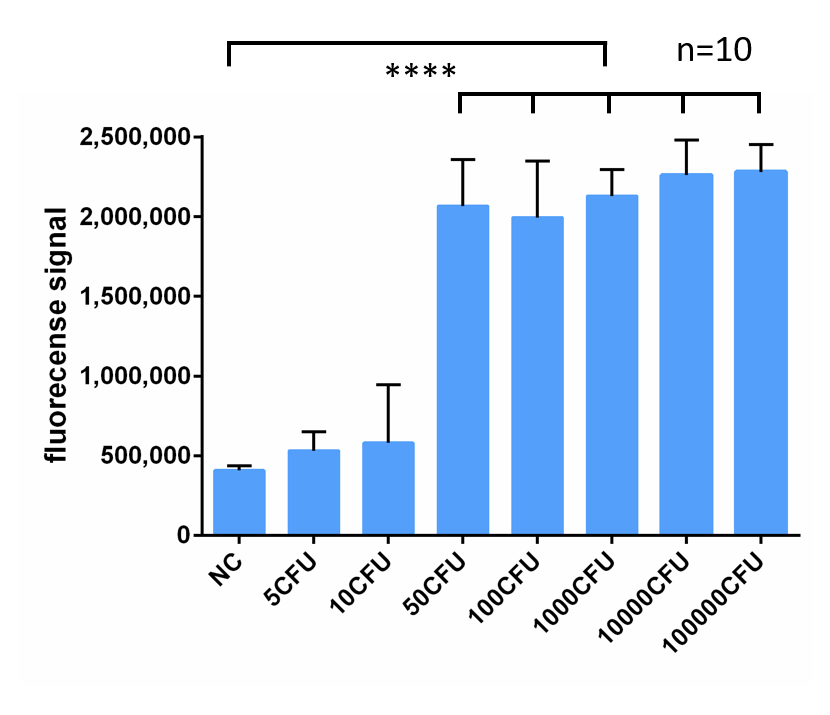

Supplement: Supplemental Material [file TEMI_A_1664939_SM9205.zip › Figure_S2_final.tif]
